# Dysglycaemia and incident aortic stenosis: a cohort study

**DOI:** 10.1136/heartjnl-2024-325150

**Published:** 2025-02-06

**Authors:** Viktor Lind, Pia Lundman, Leif Friberg, Mats Talbäck, Niklas Hammar, Göran Walldius, Mozhu Ding, Anna Norhammar

**Affiliations:** 1 Department of Clinical Sciences, Karolinska Institutet, Stockholm, Sweden; 2 Department of Cardiology, Danderyd University Hospital, Stockholm, Sweden; 3 Institute of Environmental Medicine, Karolinska Institutet, Stockholm, Sweden; 4 Division of Medicine MedS, K2, Karolinska Institute, Stockholm, Sweden; 5 Capio S:t Görans Sjukhus AB, Stockholm, Sweden

**Keywords:** aortic stenosis, diabetes mellitus, heart valve diseases, metabolic syndrome

## Abstract

**Background:**

Aortic stenosis is a degenerative condition with high mortality in its severe stages and no preventive treatment. While diabetes is a known risk factor, the role of elevated glucose levels below the diabetic threshold in the development of aortic stenosis remains unclear. This study investigated the association between dysglycaemia, including impaired fasting glucose, and the incidence of aortic stenosis.

**Methods:**

This study included 324 449 participants from the Swedish Apolipoprotein Mortality Risk cohort (1985–1996) who were free of aortic valve disease at baseline and had fasting glucose and lipid levels recorded. Participants were followed for incident aortic stenosis through the National Patient and Cause of Death Registers until 31 December 2020. Fasting glucose was categorised into low (<3.9 mmol/L), normal (3.9–6.0 mmol/L), impaired fasting glucose (6.1–6.9 mmol/L), high glucose (≥7.0 mmol/L) and diabetes. HRs were calculated using Cox proportional hazards models, with adjustments for age, sex, lipids, socioeconomic status, hypertension and kidney disease.

**Results:**

Over a mean follow-up of 25.9 years, 8523 participants developed aortic stenosis. Compared with normal glucose levels, adjusted HRs were 0.96 (95% CI 0.82 to 1.13) for low glucose, 1.36 (95% CI 1.24 to 1.50) for impaired fasting glucose, 1.79 (95% CI 1.60 to 1.99) for high glucose and 2.21 (95% CI 1.80 to 2.73) for diabetes. Spline analysis indicated a continuous increase in risk with rising glucose levels, even below the impaired fasting glucose threshold.

**Conclusions:**

Dysglycaemia, including glucose levels below the diabetic range, is associated with a higher risk of aortic stenosis.

**WHAT IS ALREADY KNOWN ON THIS TOPIC**

- Diabetes mellitus is a recognised risk factor for aortic stenosis, but the relationship between prediabetic glucose levels and aortic stenosis risk remains uncertain.

**WHAT THIS STUDY ADDS**

- This study demonstrated that the risk of developing aortic stenosis increases progressively with higher fasting glucose levels, even starting below the impaired fasting glucose threshold.
- Individuals with diabetes had the highest risk, with more than double the likelihood of aortic stenosis compared with those with normal glucose levels.

**HOW THIS STUDY MIGHT AFFECT RESEARCH, PRACTICE OR POLICY**

- These findings suggest that dysglycaemia may play a role in the pathogenesis of aortic stenosis, even at glucose levels below the diabetic threshold.
- Future studies should explore whether glucose-lowering interventions can mitigate the risk or progression of aortic stenosis.

## Introduction

 The prevalence of aortic stenosis is expected to increase with an ageing population, with previous studies showing a prevalence of 12.4% among individuals aged 75 years or above.^1^ Severe aortic stenosis has a poor prognosis, with a 5-year mortality of >60%,^2 3^ with valve replacement as the only treatment.^4^ It is projected that by 2050 the number of patients in Europe with indication for valve replacement due to aortic stenosis will have more than doubled.^1^ Globally, rheumatic fever and endocarditis as causes of aortic stenosis remains high but is decreasing, whereas in high-income countries the main cause is degenerative and due to progressive stenosis through calcification.^5^ The pathophysiology of aortic stenosis is biphasic, with an initial stage of endothelial damage and lipid infiltration followed by calcification and partly self-sustaining inflammation.^5 6^

Known risk factors for aortic stenosis include age, smoking, hypertension, dyslipidaemia, obesity, kidney disease^5 7 8^ and low socioeconomic status.^9^

In addition, persons with established diabetes are at increased risk of aortic stenosis.^8^,^11^ However, risk factors for aortic stenosis are more prevalent among patients with diabetes, which may explain part of this observation. Whether elevated fasting glucose levels affects the risk of aortic stenosis remains uncertain.

Several cardiovascular complications of diabetes slowly progress over time, with initial onset several years before diabetes diagnosis.^12^ Whether this is also true for aortic stenosis is of interest, especially for guiding possible future preventive actions. The hypothesis of this study was that the risk for aortic stenosis may be present even at glucose levels below the diagnostic threshold for diabetes.

## Methods

### Study population

**Figure 1:**
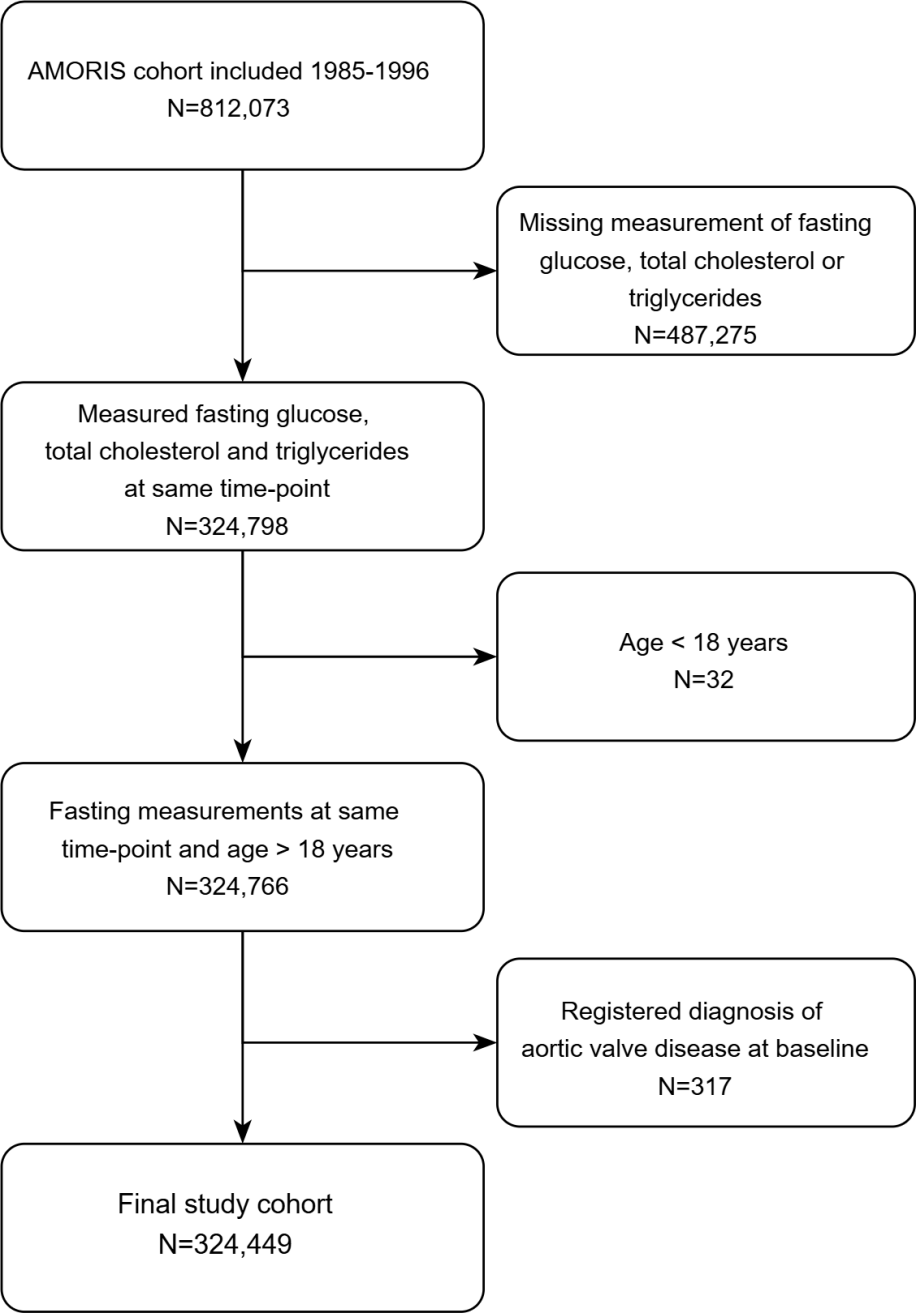
Inclusion from the original AMORIS cohort. Flow chart depicting the stepwise exclusions from the original AMORIS cohort to the final study cohort. AMORIS, Swedish Apolipoprotein Mortality Risk.

The study cohort was included from the Swedish Apolipoprotein Mortality Risk (AMORIS) cohort,^13^ consisting of 812 073 individuals, primarily from the Stockholm area, referred for laboratory testing from either occupational healthcare or primary care during the period 1985–1996. From this cohort, we included subjects confirmed to be fasting overnight prior to blood sampling, and with measured glucose, total cholesterol and triglycerides at the same examination (index examination). Subjects with diagnosed aortic valve disease (online supplemental table 1) or age below 18 years at index examination were excluded. The final study population consisted of 324 449 subjects (figure 1).

Information on comorbidities was obtained through linkage to the National Patient Register,^14^ the National Cancer Register^15^ and the National Cause of Death Register.^16^ International Classification of Diseases (ICD) codes used to identify comorbidities are presented in online supplemental table 2. Information on level of education and socioeconomic status as blue-collar or white-collar worker was obtained from the national population and housing census closest in time to the index examination up to 1990, after which socioeconomic data were obtained from the longitudinal integration database for health insurance and labour market studies. Record linkage was possible using each individual’s unique Swedish personal identification number.^17^

### Laboratory measurements

Information on analysed biomarkers was obtained from the index examination for all subjects. All blood samples were analysed in fresh blood at the CALAB medical laboratory in Stockholm, with well-documented and consistently applied analytical methods. For detailed information regarding analytical laboratory methods, see online supplemental material.

### Follow-up

Subjects were followed for incident aortic stenosis or aortic valve disease through the National Patient Register and the National Cause of Death Register using registered ICD codes. With the implementation of the ICD-10 system in 1997, aortic stenosis gained a specific ICD code. Prior to this, the ICD code for aortic valve disease was used to identify outcomes (online supplemental table 3). ICD codes were registered by physicians from inpatient or emergency care, and from 2001, also from specialised outpatient care. Mean follow-up time was 25.9 years. Subjects were followed until registered diagnosis of aortic valve disease (1985–1996) or aortic stenosis (1997–2020), death from any cause, migration or end of follow-up on 31 December 2020.

### Definitions

*Outcome* was defined as registered diagnosis of aortic valve disease in the ICD-8 or ICD-9 system, or as registered diagnosis of aortic stenosis in the ICD-10 system. *Exposure* was defined based on fasting glucose at the index examination, in subsequent analysis categorised as either low (<3.9 mmol/L), normal (3.9–6.0 mmol/L), impaired fasting glucose (IFG) (6.1–6.9 mmol/L) or high (≥7.0 mmol/L), with subjects with diagnosis of diabetes mellitus categorised separately. In addition, the defined glucose levels for IFG according to the American Diabetes Association (ADA)^18^ (5.6–6.9 mmol/L) was used for separate analysis.

*Dysglycaemia* was defined as having a fasting glucose level at or above 6.1 mmol/L or a diagnosis of diabetes. *Body mass index* (BMI) calculated as kg/m^2^ was available from the index or earlier examinations of the AMORIS cohort, the Swedish National Diabetes Register,^19^ the Swedish Medical Birth Register^20^ or research cohorts previously linked to the AMORIS cohort.^13^ Registered BMI within a timeframe of 5 years prior to or after the index examination was used, and if several measurements were available, we used that closest in time to index examination.

### Statistical analysis

Baseline characteristics were described using frequencies and percentage for categorical variables and mean and SD for continuous variables. Cox proportional regression analysis was used to estimate HRs with 95% CIs. The proportional hazards assumption for all covariates was assessed visually using log-log survival curves. Adjustments were made for age and sex, with further adjustments for total cholesterol, triglycerides, socioeconomic status, education and diagnosed hypertension and chronic kidney disease. Age-stratified analysis (age <50 years, 50–69 years and ≥70 years) was performed. Sensitivity analysis was performed in subgroups with information on BMI and apolipoprotein B (apoB)/apolipoprotein A-1 (apoA-1) ratio, respectively. To examine the association with fasting glucose as a continuous variable, HRs for fasting glucose were estimated using restricted cubic spline analysis with 95% CIs, using a glucose level of 4.2 mmol/L as the reference. Five knots were used with the first set at fasting glucose 4.0 mmol/L, the third at 4.8 mmol/L and the fifth at 6.2 mmol/L. A nested case-control study was performed to explore to what extent fasting glucose levels differed between those with incident aortic stenosis versus matched controls at different time points prior to the diagnosis. Cases were defined as all subjects with a new diagnosis of aortic valve disease or aortic stenosis during follow-up. Five controls per case was randomly selected through incidence density sampling and matched to cases by age and sex. Statistical analysis was performed using STATA V.17.0 (StataCorp, College Station, Texas, USA).

## Results

At baseline, 10 065 (3.1%) subjects had low fasting glucose, 294 671 (90.8%) had normal glucose levels, 10 353 (3.2%) had IFG, 7143 (2.2%) had high fasting glucose and 2217 (0.7%) had diagnosed diabetes. Mean age (44.8 years, total population) and the proportion of males were higher in all groups with dysglycaemia (table 1).

**Table 1:** Baseline characteristics

	N	LowN=10 065 (3.1%)	NormalN=294 671 (90.8%)	IFGN=10 353 (3.2%)	HighN=7143(2.2%)	Diagnosed DMN=2217 (0.7%)	TotalN=324 449
Age (years)	324 449	37.7 (13.3)	44.5 (13.6)	53.4 (12.3)	56.2 (11.7)	53.2 (15.2)	44.8 (13.8)
Male	324 449	4007 (39.8)	158 519 (53.8)	6964 (67.3)	4989 (69.8)	1238 (55.8)	175 717 (54.2)
Blue-collar worker	313 953	6038 (60.0)	166 249 (56.4)	6114 (59.1)	4236 (59.3)	1371 (61.8)	184 008 (56.7)
Primary education or less	271 098	2242 (22.3)	83 506 (28.3)	3875 (37.4)	2812 (39.4)	763 (34.4)	93 198 (28.7)
Occupational healthcare	324 449	6019 (59.8)	167 442 (56.8)	5218 (50.4)	3103 (43.4)	905 (40.8)	182 687 (56.3)
Hypertension	324 449	55 (0.5)	2268 (0.8)	269 (2.6)	243 (3.4)	316 (14.3)	3151 (1.0)
Valvular heart disease	324 449	3 (0)	138 (0)	9 (0.1)	9 (0.1)	9 (0.4)	168 (0.1)
IHD	324 449	147 (1.5)	7531 (2.6)	672 (6.5)	554 (7.8)	516 (23.3)	9420 (2.9)
HF	324 449	66 (0.7)	2806 (1.0)	242 (2.3)	230 (3.2)	232 (10.5)	3576 (1.1)
AF	324 449	58 (0.6)	2479 (0.8)	207 (2.0)	201 (2.8)	184 (8.3)	3129 (1.0)
Ischaemic stroke	324 449	10 (0.1)	719 (0.2)	56 (0.5)	47 (0.7)	65 (2.9)	897 (0.3)
Haemorrhagic stroke	324 449	11 (0.1)	491 (0.2)	46 (0.4)	19 (0.3)	17 (0.8)	584 (0.2)
PAD	324 449	8 (0.1)	421 (0.1)	38 (0.4)	35 (0.5)	35 (1.6)	537 (0.2)
CKD	324 449	24 (0.2)	464 (0.2)	13 (0.1)	8 (0.1)	10 (0.5)	519 (0.2)
Liver disease	324 449	47 (0.5)	1090 (0.4)	70 (0.7)	70 (1.0)	81 (3.7)	1358 (0.4)
Asthma/COPD	324 449	105 (1.0)	2263 (0.8)	122 (1.2)	93 (1.3)	71 (3.2)	2654 (0.8)
RA	324 449	76 (0.8)	1142 (0.4)	35 (0.3)	26 (0.4)	14 (0.6)	1293 (0.4)
Inflammatory disease	324 449	78 (0.8)	2257 (0.8)	100 (1.0)	80 (1.2)	63 (2.8)	2578 (0.8)
BMI	53 889	23 (3.3)	24.7 (3.7)	27.5 (4.5)	28.7 (4.9)	27.1 (5.2)	24.8 (3.8)
Glucose (mmol/L)	324 449	3.6 (0.3)	4.8 (0.5)	6.4 (0.2)	9.8 (3.2)	9.9 (4.7)	5.0 (1.2)
Fructosamine (mmol/L)	215 731	2.02 (0.2)	2.06 (0.2)	2.17 (0.2)	2.69 (0.6)	2.8 (0.6)	2.08 (0.2)
Total cholesterol (mmol/L)	324 449	5.2 (1.1)	5.6 (1.2)	6.1 (1.2)	6.1 (1.3)	5.7 (1.3)	5.6 (1.2)
Triglycerides (mmol/L)	324 449	1.00 (0.6)	1.20 (0.8)	1.86 (1.4)	2.44 (2.3)	1.84 (1.8)	1.25 (0.9)
ApoA-1 (g/L)	78 452	1.44 (0.2)	1.41 (0.2)	1.38 (0.2)	1.34 (0.2)	1.38 (0.3)	1.41 (0.2)
ApoB (g/L)	72 495	1.14 (0.3)	1.22 (0.3)	1.35 (0.4)	1.41 (0.4)	1.27 (0.4)	1.23 (0.4)
apoB/apoA-1 ratio	68 435	0.818 (0.3)	0.888 (0.3)	1.000 (0.3)	1.080 (0.3)	0.964 (0.4)	0.896 (0.3)
Creatinine (μmol/L)	303 997	78.8 (12.9)	81.6 (14.3)	85.5 (15.8)	86.3 (17.0)	87.1 (30.2)	81.8 (14.6)
Uric acid (μmol/L)	301 441	268 (69.2)	290 (70.8)	341 (80.3)	322 (86.0)	290 (96.6)	291 (72.5)
WBC (10⁹/L)	73 155	6.61 (2.4)	6.4 (2.3)	6.97 (2.1)	7.33 (3.5)	7.05 (2.0)	6.46 (2.3)
CRP (mg/L)	123 865	4.67 (10.5)	5.7 (18.8)	7.65 (24.1)	7.56 (20.9)	6.39 (11.4)	5.77 (18.8)
Haptoglobin (g/L)	260 852	1.00 (0.3)	1.06 (0.3)	1.20 (0.4)	1.23 (0.4)	1.17 (0.4)	1.07 (0.3)

Categorical variables are presented as frequencies and percentages, while continuous variables are presented as mean and SD. N represents total number of subjects with available information for each variable. Comorbidities were defined as registered corresponding ICD codes at baseline.

AF, atrial fibrillation; apolipoprotein A-1, apoA-1; apolipoprotein B, apoB; BMI, body mass index; CKD, chronic kidney disease; COPD, chronic obstructive pulmonary disease; CRP, C reactive protein; DM, diabetes mellitus; HF, heart failure; IFG, impaired fasting glucose; IHD, ischaemic heart disease; PAD, peripheral arterial disease; RA, rheumatoid arthritis; WBC, white blood cell.

Apart from chronic kidney disease and rheumatoid arthritis, the presence of comorbidities was higher among subjects with dysglycaemia, especially cardiovascular disease. Among those with diagnosed diabetes at baseline, the prevalence of a hospital diagnosis of hypertension, ischaemic heart disease, heart failure and atrial fibrillation was clearly higher than in subjects with normal glucose levels (14%, 23%, 11% and 8% vs 0.8%, 3%, 2% and 0.8%).

Total cholesterol, triglyceride levels and apoB/apoA-1 ratio was higher in all groups with dysglycaemia. Similarly, in these groups BMI was higher, however information on BMI was only available in 17% of all subjects (n=53 899).

### Outcome

During a mean follow-up of 25.9 years, 8523 events of aortic stenosis occurred, resulting in a cumulative incidence of 2.6%. Of these, 897 (10.5%) were diagnosed with aortic valve disease before the implementation of ICD-10 in 1997. An association with incident aortic stenosis was seen in all groups with dysglycaemia with an HR (95% CI) of 1.36 (1.24 to 1.5), 1.79 (1.60 to 1.99) and 2.21 (1.80 to 2.73) for subjects with IFG, high glucose and diagnosed diabetes, respectively (table 2).

**Table 2:** HRs for aortic stenosis stratified by fasting glucose.

Glucose levels	Subjects	Events	Events/10 000 person-years(95% CI)	Absolute risk (%)	HR adjusted for age and sex	Further adjusted for SEI, TC and TG	Further adjusted forhypertension and CKD
Low	10 065(3.1%)	150	5.4(4.6 to 6.3)	1.5	0.92(0.78 to 1.08)	0.96(0.82 to 1.13)	0.96(0.82 to 1.13)
Normal	294 671(90.8%)	7437	9.6(9.4 to 9.8)	2.5	1 (ref)	1 (ref)	1 (ref)
IFG	10 353(3.2%)	460	20.6(18.7 to 22-5)	4.4	1.43(1.30 to 1.58)	1.37(1.25 to 1.51)	1.36(1.24 to 1.5)
High	7143(2.2%)	384	29.0(26.2 to 32.0)	5.4	1.93(1.74 to 2.14)	1.80(1.61 to 2.0)	1.79(1.60 to 1.99)
DM	2217(0.7%)	92	23.6(19.2 to 28.9)	4.1	2.34(1.90 to 2.87)	2.37(1.93 to 2.91)	2.21(1.80 to 2.73)

Observed events, incidence rate per 10 000 person-years and HR for incident aortic stenosis.

CKD, chronic kidney disease; DM, diabetes mellitus; IFG, impaired fasting glucose; ref, reference; SEI, socioeconomic index including education; TC, total cholesterol; TG, triglycerides.

Subjects who developed aortic stenosis during follow-up had a higher mean age at baseline (54.4 vs 44.6) and a higher proportion of males, as well as higher fasting glucose, lipid levels, apoB/apoA-1 ratio and CRP.

**Figure 2:**
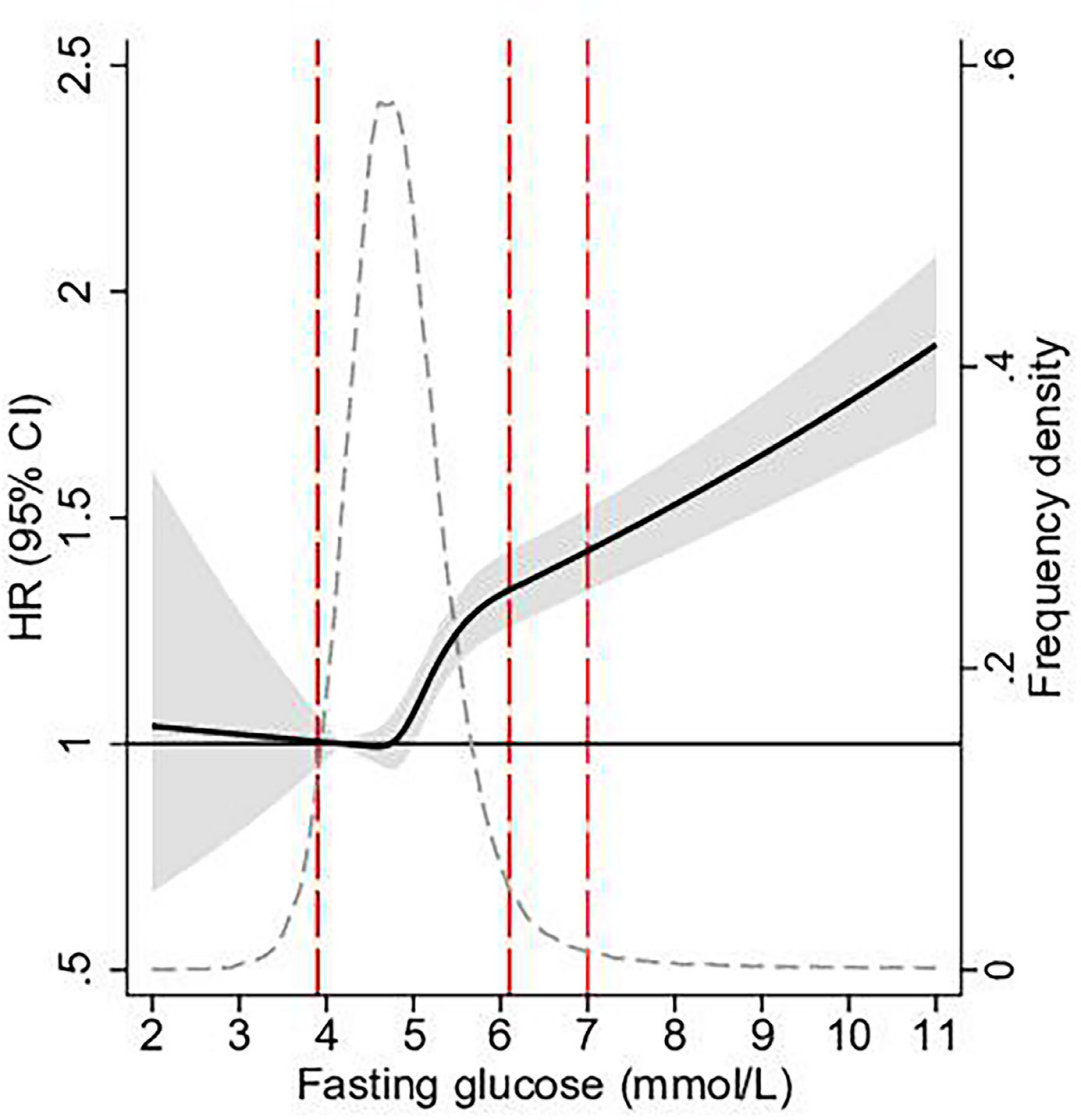
HR for aortic stenosis by fasting glucose. HRs (black line) with 95% CIs (grey area) for incident aortic stenosis by fasting glucose presented as splines, adjusted for age, sex, total cholesterol and triglycerides, with glucose 4.2 mmol/L set as reference. Subjects with fasting glucose <5 SD or >5 SD not presented. Grey dotted line presents proportion of study cohort with corresponding fasting glucose at baseline (frequency density). Red dotted line presents limits for low glucose and lower and upper limit for impaired fasting glucose, respectively.

Cubic spline analysis showed a continuously stronger association with aortic stenosis with increasing glucose levels, starting already below the diagnostic threshold for IFG (6.1 mmol/L) (figure 2).

When comparing ADA criteria for IFG with that of the WHO criteria, further analysis with IFG categorised according to the definition by the ADA (5.6–6.9 mmol/L) resulted in an additional 23 374 subjects categorised as having IFG (n=33 727). For this group, the observed HR showed a similar pattern to those categorised according to the WHO criteria, although slightly lower (HR 1.24 vs 1.36) (online supplemental table 4).

In the nested case-control analysis, higher levels of glucose in cases compared with controls were consistently present >30 years prior to the diagnosis of aortic stenosis and became more pronounced closer in time to the diagnosis (online supplemental figure 1).

When exploring the association by age groups, the association were strongest among those at younger age at inclusion (online supplemental table 5). For the oldest age group (≥70 years at inclusion), the association showed a similar pattern to the overall analysis, but the association with IFG no longer remained statistically significant.

### Subgroup analysis

Subgroup analysis among subjects with available apoB/apoA-1 ratio (n=68 435) showed an increased association with aortic stenosis among all groups with elevated glucose, with the highest association observed in those with known diabetes. Adjustment for the apoB/apoA-1 ratio did not affect this association among those with diabetes and only slightly attenuated the association for those with IFG and high glucose (online supplemental table 6).

Further subgroup analysis of subjects with available data on BMI (n=53 889, baseline characteristics in online supplemental table 7) showed a strong association with aortic stenosis for those with high glucose levels or diagnosed diabetes. These associations appeared to be somewhat attenuated after adjusting for BMI (HR 1.72 and 2.59 for high glucose and diabetes, respectively). For IFG, the association was weaker and the point estimate after adjustment for BMI suggested no association with aortic stenosis, although CIs were wide and overlapping (online supplemental table 8).

## Discussion

In this large population-based Swedish cohort followed for a mean of 26 years, there are two main findings. First, there is an association between dysglycaemia and aortic stenosis, both overall and after adjustments for important risk factors such as hyperlipidaemia and hospital diagnoses of hypertension or kidney disease. Second, elevated fasting glucose is continuously associated with incident aortic stenosis. This association showed a dose-response relationship, present even below both the ADA and WHO definitions of prediabetes. A similar association between glucose levels and other cardiovascular outcomes has previously been shown, even before the established diagnosis of diabetes.^12^

The association with aortic stenosis was strongest for those with known diabetes at baseline, with more than twice the risk for aortic stenosis compared with subjects with normal glucose levels, which is similar to results from previous observational studies.^8^ Subjects with high glucose had the highest absolute risk of aortic stenosis, which could in part be due to a slightly higher mean age at baseline.

### Lipids and apolipoproteins

Elevated low-density lipoprotein (LDL) cholesterol, decreased levels of apoA-1 and elevated levels of apoB are associated with increased risk of aortic stenosis.^21 22^ Statin treatment has not been shown to be effective in reducing this risk.^23 24^ However, a secondary analysis of the SEAS study, evaluating simvastatin and ezetimibe combined, showed a 60% reduced risk for valve replacement among patients with mild aortic stenosis and LDL >4 mmol/L.^25^ Additionally, there are ongoing trials studying the effect of other lipid-lowering treatments on the progression of aortic stenosis.^26^ In our study, information on apolipoprotein levels was available in a subgroup (n=68 435), and adjusting for apoB/apoA-1 ratio on top of total cholesterol and triglycerides had essentially no effect on the association between dysglycaemia and aortic stenosis.

### Pathogenesis of aortic stenosis and effect of dysglycaemia

The development of degenerative aortic stenosis is initiated through endothelial damage and successive lipid infiltration and inflammation.^5^ This in turn causes the localised deposition of calcium crystals, which maintains an inflammatory response and progresses the disease into a second fibrotic phase. At this stage, the disease progression is driven by fibrosis and calcification, causing osteogenic processes resulting in pronounced calcification and loss of function of the aortic valve.^6^ Several mechanisms through which diabetes may drive progression to aortic stenosis has been suggested. When examined ex vivo, aortic valves from patients with diabetes have increased levels of advanced glycated end-products, in turn associated with a smaller aortic valve area, indicating more pronounced stenosis.^27^ Furthermore, ex vivo studies have shown higher expression of nuclear factor kappa B and bone morphogenetic protein 2, which are involved in the progression of aortic stenosis, in aortic valves from individuals with diabetes.^28^

Few studies have examined the association of glucose levels with incident aortic stenosis. A large observational study investigating the risk for valvular heart disease among patients with type 2 diabetes found that although diabetes increased the risk for aortic stenosis, higher haemoglobn A1c (HbA1c) did not affect this risk.^9^ It has been suggested that metabolic risk factors mainly influence the initial phase in the establishment of aortic stenosis, and less in the continued progression of the disease.^11^ The initial establishment of degenerative aortic stenosis is characterised by endothelial damage and inflammation.^26^ Hyperglycaemia and prediabetes are associated with increased levels of inflammatory markers and cause an upregulation of pro-inflammatory cellular pathways, leading to increased oxidative stress.^29^ After the establishment of initial calcification however, continued disease progression becomes, in part, self-propagating.^6^ Thus, elevated glucose levels may influence the establishment of aortic stenosis, and it is possible that this occurs already at lower glucose levels and before the diagnosis of diabetes. This would be consistent with our findings of an association already at prediabetic levels of glucose. Furthermore, the nested case-control study showed that, decades prior to diagnosis, subjects with incident aortic stenosis had higher fasting glucose levels than controls.

### Body mass index

Information on BMI was only available for a subgroup (n=53 889), in which IFG was not shown to be associated with aortic stenosis. This subgroup did differ from the total study cohort in some respects, however, particularly in having a lower prevalence of cardiovascular comorbidity and a higher proportion referred via occupational healthcare (80% vs 56% in the total cohort), suggesting that subjects with measured BMI may have been healthier at baseline.

Adjusting for BMI attenuated the risk for aortic stenosis among those with high glucose levels and diagnosed diabetes. Due to the small size of this subgroup, these results must be interpreted with caution, but this suggests that BMI remains a potential confounder. A recent study on Swedish patients with diabetes did indeed find BMI to be associated with aortic stenosis.^9^

With the expanding role of novel glucose-lowering drugs in the treatment of other cardiovascular diseases, there are opportunities to study whether these may also have a preventive effect on aortic stenosis. Glucagon-like peptide-1 receptor agonists, which lower blood glucose and facilitate weight loss, may be of particular interest. Given the biphasic pathophysiology of aortic stenosis, which involves initial inflammation and accumulation of lipoproteins, it is possible that treatment of metabolic risk factors may have a preventive effect, particularly in the early stages of the disease.

### Strengths and limitations

The main strengths of this study are its large size and long follow-up of this population-based cohort, along with a wide array of biochemical analyses performed at baseline. All analyses were conducted on fresh blood samples in the same laboratory, ensuring consistency and comparability of the biomarker information. Furthermore, most subjects were referred for testing through routine health check-ups via occupational healthcare, and the overall prevalence of comorbidities was low, with no subjects hospitalised at the time of index health examination. Linkage to several high-quality national registries enabled precise follow-up and provided additional information, such as prevalent comorbidities and socioeconomic status.

The limitations of this study are mainly due to the registry-based methodology, which resulted in a lack of information on lifestyle factors, including smoking status. Additionally, we did not have information on blood pressure or pharmacological therapy at baseline. Therefore, information on hypertension at baseline was defined as a registered diagnosis of hypertension in the National Patient Register. Although the ICD system has high validity in the National Patient Register, it has previously been shown to have limited coverage for hypertension specifically.^14 30^ Thus, the prevalence of hypertension in our cohort has likely been substantially underestimated and remains a potential confounder. Furthermore, HbA1c was not included, as it was not routinely analysed at the time of inclusion and was largely unavailable. Since HbA1c reflects prolonged exposure to elevated glucose levels, it is possible that elevated HbA1c could show a stronger association with incident aortic stenosis. It is possible that we would have identified more subjects as having dysglycaemia if HbA1c had been available, meaning the association based on fasting glucose levels could be underestimated. As HbA1c has become widely used in clinical practice to detect dysglycaemia, this is an important area for further studies.

In a Swedish setting, the diagnosis of aortic stenosis is based on echocardiographic findings, with the examination typically referred either due to the auscultation of a heart murmur or as part of an evaluation for symptoms such as breathlessness, chest pain, syncope or signs of congestive heart failure. Echocardiography is not performed without prior suspicion of cardiovascular disease, and we do not believe that subjects with elevated fasting glucose received echocardiography more frequently than those with normal glucose levels. However, it is possible that subjects with a diagnosis of diabetes were monitored more regularly, which could contribute to a higher incidence of diagnosed aortic stenosis in this group.

## Conclusion

This long-term, large population-based study showed that dysglycaemia, including both diabetes and prediabetes, is associated with an increased risk for the future development of aortic stenosis. We found fasting glucose to be associated with incident aortic stenosis even below the standard diabetes threshold, indicating that glucose is a continuous risk factor.

## Supplementary material

10.1136/heartjnl-2024-325150 online supplemental file 1

## Data Availability

Data are available on reasonable request.
