# Supplementary material for: Dysglycaemia and incident aortic stenosis: a cohort study
Source: Heart. 2025 Feb 6;111(14):e325150. doi: 10.1136/heartjnl-2024-325150 (PMC12229057; doi:10.1136/heartjnl-2024-325150)

**Supplementary materials**

**Laboratory measurements and analytical methods**

Glucose was analysed from serum enzymatically with a glucose oxidase/peroxidase method based on a technique as per the GOD-PAP method using automated multichannel analysers. Levels of serum glucose were standardized to international standards of plasma glucose. Fructosamine levels were analysed using the Nitroblue Teterazolium (NBT) colorimetric technique using the same automatic multichannel analysers. Triglycerides and total cholesterol were analysed through enzyme techniques. Levels of apoB and apoA-1 was assessed through immunoturbidimetry. Creatinine levels were analysed with the non-kinetic alkaline picrate method (Jaffe´), from 1985 through 1992 by AutoChemistPRISMA and from 1993 through 1996 by DAX-96 analyser. Haemoglobin and white blood cells were measured by flow cytometry from whole blood with CoulterR STKS Haematology System (Coulter Corporation). CRP and haptoglobin was analysed with immunoturbi-dimetric assay.

**Supplementary table 1** - International Classification of Diseases [ICD] codes 8/9/10 used to define previous aortic valve disease as exclusion criteria.

|  | ICD-9 | ICD-8 |
| --- | --- | --- |
| Aortic valve disease | 395-396, 424.1 (424B), 746.3 (746D), 746.4 (746E) | 395, 396, 424.9, 746.6 |

**Supplementary table 2** - International Classification of Diseases [ICD] code 8/9/10 diagnoses and Classification of Surgical Procedures NOMESCO (Nordic Medico-Statistical Committee) codes used to define comorbidities.

|  | ICD-10 | ICD-9 | ICD-8 |
| --- | --- | --- | --- |
| Ischemic heart disease | I20-I25 | 410-414 | 410-434 |
| Heart failure | I50 | 428 | 427-429 |
| Atrial fibrillation | I48 | 427.3 (427D) | 427.9 |
| Stroke - Ischaemic | I63-I64, I69.3-I69.4 | 433-434 | 432, 433, 434, 437 |
| Stroke - Hemorragic | I60-I62, I69.0-I69.2 | 430-432 | 430-431 |
| Asthma/COPD | J43-46 | 491, 492, 493, 496 | 490-493 |
| Liver disease | K70-77 | 570 – 573 | 570 - 573 |
| Chronic kidney disease | N18 | 585 | 582, 792, Y29.08 |
| History of cancer | C-chapter | 140 – 165, 170-175, 179-208 | 140-163, 170-174, 180-207 |
| Diabetes | E10, E11, E13, E14 | 250 | 250 |
| Hypertension | I10-I15 | 401-405 | 400-404 |
| Other valvular heart disease | I05, I07-I09, I34, I36, I37 | 394, 397, 398, 424.0 (424A), 424.2(424C), 424.3(424D) | 394, 397, 398, 424.0, 424.9 |
| Peripheral arterial disease | I70, I73.8, I73.9 | 440, 443 | 440, 443 |
| Rheumatoid arthritis | M05, M06 | 714 | 712 |
| Systemic inflammatory disease | M30-M36 | 446, 710, 725 | 446, 734, 716 |
| Ankylosing spondylitis | M45 | 720.0 (720A), 720.9 (720X), | 712.4 |
| Psoriasis | L40 | 696.0, 696.1 (696A, 696B) | 696.0, 696.1 |

**Supplementary table 3** - International Classification of Diseases [ICD] code 9/10 and Classification of Surgical Procedures NOMESCO (Nordic Medico-Statistical Committee) codes for diagnoses used to define events.

|  | ICD-10 | ICD-9 | Procedural code |
| --- | --- | --- | --- |
| Aortic stenosis | I35.0, I35.2 |  |  |
| Aortic valve disease |  | 424.1 |  |
| Aortic valve replacement |  |  | FMD (ICD 10) |

**Supplemetary table 4** - Events and HR:s for aortic valve disease/aortic stenosis with IFG defined by ADA-criteria (5.6-6.9 mmol/L)

| Glucose levels | Subjects | Events | Events/10,000 person-years  (95% CI) | HR adj for age and sex | HR further adj for SEI, TC and TG | HR further adj for hypertension and CKD |
| --- | --- | --- | --- | --- | --- | --- |
| Low | 10,065  (3.1%) | 150 | 5.4  (4.6-6.3) | 0.94  (0.80-1.11) | 0.98  (0.83-1.15) | 0.98  (0.83-1.15) |
| Normal | 271,297  (83.6%) | 6,546 | 9.1  (8.9-9.3) | 1 (ref) | 1 (ref) | 1 (ref) |
| IFG by ADA | 33,727  (10.4%) | 1,351 | 17.4  (16.5-18.4) | 1.31  (1.23-1.39) | 1.25  (1.18-1.32) | 1.24  (1.17-1.32) |
| High | 7,143  (2.2%) | 384 | 29.0  (26.2-32.0) | 1.98  (1.79-2.20) | 1.84  (1.65-2.05) | 1.83  (1.64-2.04) |
| DM | 2,217  (0.7%) | 92 | 23.6  (19.2-28.9) | 2.40  (1.95-2.95) | 2.42  (1.97-2.98) | 2.26  (1.84-2.79) |

Abbreviations: IFG=Impaired fasting glucose. ADA=American Diabetes Association. DM=Diabetes mellitus. SEI=Socioeconomic index incl. Education. TC=Total cholesterol. TG=Triglycerides. CKD=Chronic kidney disease

**Supplementary figure 1** – Nested case control analysis showing index fasting glucose of cases vs controls. Five controls per case was randomly selected through incidence density sampling and matched to cases by age and sex. Throughout follow-up cases had higher index glucose levels compared to controls, with a more pronounced difference among cases with diagnosed aortic stenosis close in time from baseline.


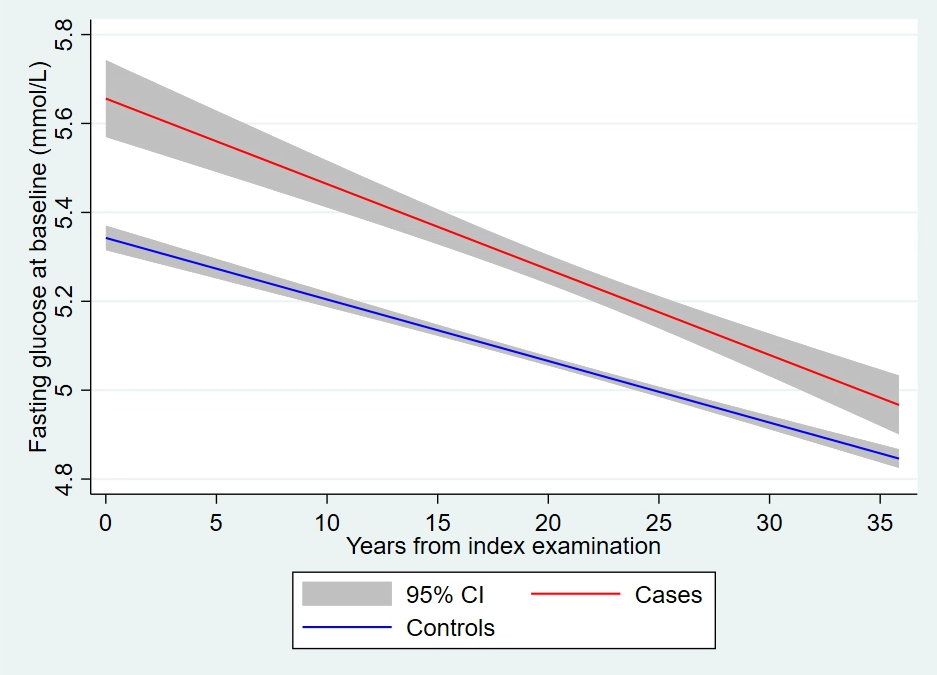


**Supplementary table 5** - Events and HR:s for aortic valve disease/aortic stenosis stratified by fasting glucose and age at index examination.

| Age group | Glucose levels | Subjects | Events | HR adj for age and sex | further adj for SEI, TC and TG | further adj for  hypertension and CKD |
| --- | --- | --- | --- | --- | --- | --- |
| <50 years | Low | 8,123  (3.9%) | 56 | 0.82  (0.63-1.07) | 0.86  (0.66-1.12) | 0.86  (0.66-1.12) |
|  | Normal | 191,853  (92.8%) | 2,388 | 1 (ref) | 1 (ref) | 1 (ref) |
|  | IFG | 3,872  (1.9%) | 105 | 1.66  (1.36-2.02) | 1.53  (1.26-1.87) | 1.52  (1.25-1.85) |
|  | High | 1,979  (1.0%) | 80 | 2.73  (2.18-3.41) | 2.21  (1.74-2.81) | 2.19  (1.72-2.78) |
|  | DM | 898  (0.4%) | 24 | 3.08  (2.06-4.61) | 3.10  (2.07- 4.63) | 2.86  (1.91-4.30) |
| 50-69 years | Low | 1,763  (1.7%) | 84 | 0.98  (0.79-1.22) | 1.01  (0.82-1.26) | 1.01  (0.82-1.26) |
|  | Normal | 91,025  (88.0%) | 4,492 | 1 (ref) | 1 (ref) | 1 (ref) |
|  | IFG | 5,442  (5.3%) | 311 | 1.44  (1.28-1.62) | 1.38  (1.23-1.55) | 1.38  (1.23-1.55) |
|  | High | 4,253  (4.1%) | 257 | 1.85  (1.63-2.10) | 1.75  (1.54-2.0) | 1.74  (1.53-1.99) |
|  | DM | 912  (0.9%) | 56 | 2.44  (1.87-3.18) | 2.41  (1.85- 3.14) | 2.24  (1.72-2.93) |
| ≥70 years | Low | 179  (1.3%) | 10 | 1.42  (0.76-2.66) | 1.47  (0.79-2.75) | 1.47  (0.78-2.74) |
|  | Normal | 11,793  (82.7%) | 557 | 1 (ref) | 1 (ref) | 1 (ref) |
|  | IFG | 1,039  (7.3%) | 44 | 1.04  (0.76-1.41) | 1.05  (0.77-1.44) | 1.05  (0.77-1.44) |
|  | High | 911  (6.4%) | 47 | 1.53  (1.36-2.07) | 1.60  (1.18-2.18) | 1.60  (1.17-2.17) |
|  | DM | 339  (2.4%) | 12 | 1.41  (0.79-2.50) | 1.51  (0.85- 2.7) | 1.51  (0.84-2.69) |

Abbreviations: IFG=Impaired fasting glucose. ADA=American Diabetes Association. DM=Diabetes mellitus. SEI=Socioeconomic index incl. Education. TC=Total cholesterol. TG=Triglycerides. CKD=Chronic kidney disease

**Supplementary table 6 -** Events and HR:s for aortic valve disease/aortic stenosis stratified by fasting glucose for subgroup with measured Apolipoprotein A1 and -B at index examination.

| Glucose levels | Subjects | Events | Events/10,000 person-years  (95% CI) | HR adj for age and sex | HR further adj for apoB/A1-ratio | HR adj for age, sex, SEI, TC and TG | HR further adj for apoB/A1-ratio |
| --- | --- | --- | --- | --- | --- | --- | --- |
| Low | 1,593  (2.3%) | 29 | 6.8  (4.7-9.8) | 0.87  (0.60-1.25) | 0.89  (0.62-1.29) | 0.91  (0.63-1.32) | 0.91  (0.63-1.32) |
| Normal | 61,851  (90.4%) | 1,691 | 10.9  (10.4-11.4) | 1 (ref) | 1 (ref) | 1 (ref) | 1 (ref) |
| IFG | 2,683  (3.9%) | 117 | 20.6  (17.2-24.7) | 1.35  (1.12-1.63) | 1.30  (1.08-1.57) | 1.27  (1.05-1.54) | 1.27  (1.05-1.54) |
| High | 1,720  (2.5%) | 101 | 31.8  (26.2-38.7) | 1.92  (1.57-2.35) | 1.76  (1.44-2.16) | 1.73  (1.40-2.13) | 1.72  (1.40-2.12) |
| DM | 588  (0.9%) | 39 | 40.8  (29.8-55.8) | 3.26  (2.37-4.49) | 3.16  (2.29-4.34) | 3.27  (2.37-4.51) | 3.26  (2.36-4.49) |

Abbreviations: IFG=Impaired fasting glucose. ADA=American Diabetes Association. DM=Diabetes mellitus. SEI=Socioeconomic index incl. Education. TC=Total cholesterol. TG=Triglycerides.

**Supplementary table 7 –** Baseline characteristics for subgroup with registered BMI

|  | **N** | **Low**  n=1,559 (2.9%) | **Normal**  n=49,122 (91.2%) | **IFG**  n=1,906 (3.5%) | **High**  n=982  (1.8%) | **Diagnosed DM**  n=320 (0.6%) | **Total**  n=53,889 |
| --- | --- | --- | --- | --- | --- | --- | --- |
| Age (years) | 53,889 | 37.4 (12.5) | 43.6 (12.1) | 50.7 (10.5) | 53 (9.2) | 51 (11.9) | 43.9 (12.2) |
| Female | 53,889 | 875 (56.1) | 19,825 (40.4) | 477 (25.0) | 223 (22.7) | 113 (35.3) | 21,513 (39.9) |
| Blue-collar worker | 53,889 | 1,013 (65.0) | 29,739 (60.5) | 1,201 (63.0) | 634 (64.6) | 205 (64.1) | 32,792 (60.9) |
| Primary education or less | 53,889 | 382 (24.5) | 15,183 (30.9) | 788 (41.3) | 421 (42.9) | 116 (36.3) | 16,890 (31.3) |
| Occupational healthcare | 53,889 | 1,158 (74.3) | 39,223 (79.8) | 1,605 (84.2) | 785 (79.9) | 261 (81.6) | 43,032 (79.9) |
| Hypertension | 53,889 | 9 (0.6) | 354 (0.7) | 38 (2.0) | 34 (3.5) | 49 (15.3) | 484 (0.9) |
| Valvular heart disease | 53,889 | 1 (0.1) | 16 (0) | 1 (0.1) | 0 (0) | 2 (0.6) | 20 (0) |
| IHD | 53,889 | 19 (1.2) | 1,042 (2.1) | 90 (4.7) | 58 (5.9) | 61 (19.1) | 1,270 (2.4) |
| HF | 53,889 | 12 (0.8) | 365 (0.7) | 30 (1.6) | 32 (3.3) | 20 (6.3) | 459 (0.9) |
| AF | 53,889 | 12 (0.8) | 323 (0.7) | 28 (1.5) | 27 (2.7) | 15 (4.7) | 405 (0.8) |
| Ischemic stroke | 53,889 | 1 (0.1) | 125 (0.3) | 7 (0.4) | 3 (0.3) | 5 (1.6) | 141 (0.3) |
| Hemorragic stroke | 53,889 | 2 (0.1) | 79 (0.2) | 8 (0.4) | 1 (0.1) | 1 (0.3) | 91 (0.2) |
| PAD | 53,889 | 2 (0.1) | 63 (0.1) | 5 (0.3) | 4 (0.4) | 4 (1.3) | 78 (0.1) |
| CKD | 53,889 | 0 (0) | 75 (0.2) | 3 (0.2) | 2 (0.2) | 0 (0) | 80 (0.1) |
| Liver disease | 53,889 | 11 (0.7) | 150 (0.3) | 3 (0.2) | 7 (0.7) | 4 (1.3) | 175 (0.3) |
| Asthma/COPD | 53,889 | 17 (1.1) | 387 (0.8) | 14 (0.7) | 12 (1.2) | 14 (4.4) | 444 (0.8) |
| RA | 53,889 | 7 (0.4) | 187 (0.4) | 7 (0.4) | 5 (0.5) | 2 (0.6) | 208 (0.4) |
| Inflammatory disease | 53,889 | 11 (0.7) | 362 (0.7) | 21 (1.1) | 10 (1.0) | 8 (2.5) | 412 (0.8) |
| Glucose (mmol/L) | 53,889 | 3.58 (0.3) | 4.8 (0.5) | 6.4 (0.2) | 9.5 (2.9) | 9.9 (4.4) | 5.0 (1.1) |
| Fructosamine (mmol/L) | 35,057 | 2.03 (0.2) | 2.07 (0.2) | 2.18 (0.2) | 2.65 (0.6) | 2.67 (0.6) | 2.08 (0.2) |
| Total cholesterol (mmol/L) | 53,889 | 5.2 (1.1) | 5.6 (1.1) | 6.0 (1.2) | 6.1 (1.4) | 5.6 (1.2) | 5.6 (1.1) |
| Triglycerides (mmol/L) | 53,889 | 1.02 (0.7) | 1.23 (0.8) | 1.95 (1.7) | 2.45 (2.3) | 1.83 (1.6) | 1.28 (1.0) |
| Apolipoprotein A-1 (g/L) | 21,288 | 1.44 (0.3) | 1.39 (0.2) | 1.37 (0.2) | 1.33 (0.2) | 1.38 (0.3) | 1.39 (0.2) |
| Apolipoprotein B (g/L) | 19,157 | 1.09 (0.3) | 1.14 (0.3) | 1.25 (0.4) | 1.31 (0.4) | 1.14 (0.4) | 1.15 (0.3) |
| apoB/apoA-1 ratio | 18,956 | 0.782 (0.3) | 0.842 (0.3) | 0.928 (0.3) | 1.00 (0.3) | 0.852 (0.3) | 0.849 (0.3) |
| Creatinine (umol/L) | 42,502 | 78.3 (12.9) | 81.2 (13.5) | 84.9 (14.7) | 85.3 (15.9) | 85.5 (26.2) | 81.3 (13.7) |
| Uric acid (umol/L) | 43,734 | 270 (71.5) | 291 (68.3) | 342 (77.0) | 316 (80.8) | 290 (94.1) | 292 (69.9) |
| WBC (10⁹/L) | 3,537 | 6.74 (3.5) | 6.35 (2.0) | 6.71 (2.0) | 7.41 (2.4) | 7.37 (2.2) | 6.40 (2.1) |
| CRP (mg/L) | 23,211 | 4.24 (6.3) | 5.64 (22.1) | 6.88 (20.0) | 6.92 (24.1) | 6.18 (9.4) | 5.65 (21.7) |
| Haptoglobin (g/L) | 36,696 | 0.981 (0.3) | 1.04 (0.3) | 1.16 (0.3) | 1.21 (0.3) | 1.13 (0.4) | 1.05 (0.3) |

*Categorical variables presented as frequencies and percentages, continuous variables presented as mean and standard deviation (SD). N represents total number of subjects with available information for each variable. Co-morbidities were defined as registered corresponding ICD-codes at baseline. Abbreviations: PAD=Peripheral arterial disease. CKD=Chronic kidney disease. COPD=Chronic obstructive pulmonary disease. RA=Rheumatoid arthritis. WBC=White blood cell-count. CRP=C-Reactive Protein.*

**Supplementary table 8** - Events and HR:s for aortic valve disease/aortic stenosis stratified by fasting glucose for subgroup with measured BMI within 5 years from index examination.

| Glucose levels | Subjects | Events | Events/10,000 person-years  (95% CI) | HR adj for age and sex | HR further adj for BMI | HR adj for age, sex, SEI, TC and TG | HR further adj for BMI |
| --- | --- | --- | --- | --- | --- | --- | --- |
| Low | 1,559  (2.9%) | 20 | 4.6  (3.0-7.1) | 0.74  (0.47-1.14) | 0.80  (0.51-1.24) | 0.76  (0.49-1.18) | 0.82  (0.53-1.27) |
| Normal | 49,122  (91.2%) | 1,179 | 9.1  (8.6-9.7) | 1 (ref) | 1 (ref) | 1 (ref) | 1 (ref) |
| IFG | 1,906  (3.5%) | 66 | 15.3  (12.0-19.5) | 1.22  (0.95-1.57) | 1.02  (0.79-1.31) | 1.14  (0.89-1.47) | 0.99  (0.77-1.28) |
| High | 982  (1.8%) | 60 | 30.1  (23.4-38.8) | 2.35  (1.81-3.05) | 1.84  (1.41-2.40) | 2.04  (1.55-2.68) | 1.72  (1.31-2.25) |
| DM | 320  (0.6%) | 19 | 30.5  (19.4-47.8) | 3.04  (1.93-4.79) | 2.47  (1.57-3.90) | 3.07  (1.94-4.84) | 2.59  (1.64-4.10) |

Abbreviations: IFG=Impaired fasting glucose. ADA=American Diabetes Association. DM=Diabetes mellitus. SEI=Socioeconomic index incl. Education. TC=Total cholesterol. TG=Triglycerides.

**Supplementary figure 2** – Kaplan-Meier curve of event-free survival by fasting glucose at baseline


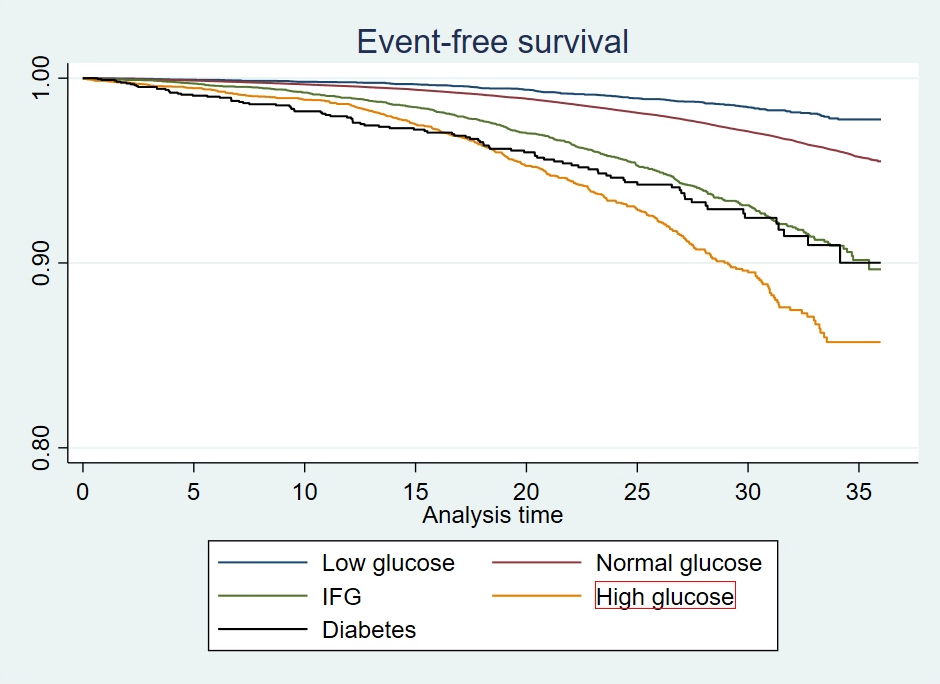

Supplement: online supplemental file 1 [file heartjnl-111-14-s001.docx]
